# Small lung lesions invisible under fluoroscopy are located accurately by three-dimensional localization technique on chest wall surface and performed bronchoscopy procedures to increase diagnostic yields

**DOI:** 10.1186/s12890-016-0328-8

**Published:** 2016-11-29

**Authors:** Chaosheng Deng, Xiaoming Cao, Dawen Wu, Haibo Ding, Ruixiong You, Qunlin Chen, Linying Chen, Xin Zhang, Qiaoxian Zhang, Yongquan Wu

**Affiliations:** 1 Division of Respiratory and Critical Care Medicine, First Affiliated Hospital of Fujian Medical University, Fuzhou, 350005 Fujian China; 2 Department of Respiratory Disease, First Hospital of Longyan City, 364000 Fujian, China; 3 Department of Medical Imaging, First Affiliated Hospital of Fujian Medical University, Fuzhou, 350005 Fujian China; 4 Department of Pathology, First Affiliated Hospital, Fujian Medical University, Fuzhou, Fujian Province China

**Keywords:** Small peripheral pulmonary lesions (PPLs), Invisible under fluoroscopy, Three-dimensional localization technique, Opaque soft copper wires, Bronchoscopy, Diagnostic yields, Radial endobronchial ultrasound (R-EBUS), Rapid on-site evaluation (ROSE)

## Abstract

**Background:**

Nowadays, small peripheral pulmonary lesions (PPLs) are frequently detected and the prognosis of lung cancer depends on the early diagnosis. Because of the high fee and requiring specialized training, many advanced techniques are not available in many developing countries and rural districts.

**Methods:**

Three sets of opaque soft copper wires visible under the fluoroscopy (Flu) in the Flu-flexible bronchoscopy (FB) group (*n* = 24), which determined the three planes of the lesion, were respectively placed firmly on the surface of the chest wall with adhesive tape on the chest wall. The FB tip was advanced into the bronchus toward the crosspoint of the three perpendicular planes under Flu with careful rotation of a C-arm unit. Then the specimen were harvested focusing around the crosspoint for pathologic diagnosis. The rapid on-site evaluation (ROSE) procedure was also performed. The average Flu time during FB procedures were recorded and diagnostic accuracy rates in the Flu-FB group were compared with the other group guided by radial endobronchial ultrasound (R-EBUS) (*n* = 23).

**Results:**

The location of the core point of the lesion, whether it was visible or not under the fluoroscopy could be recognized by three-dimensional localization technique. The accuracy rates of diagnostic yields were 62.5% in the Flu-FB group, and was similar as 65.2% in the R-EBUS group (*P* > 0.05). However, in the Flu-FB group, there was a decreasing tendency on accurate diagnosis rates of lower lobe (LL) lesions when comparing with non-LL lesions (3/8 = 37.5% vs 12/16 = 75%, *P* = 0.091) while in the R-EBUS group it was similar (9/12 = 75% vs 6/11 = 54.6%, *P* = 0.278). In the Flu-FB group, fluoroscopy time was negatively correlated with the lesion length (*r* = −0.613, *P* = 0.001), however, there was no significant difference between the lesions invisible or not (5.83 ± 1.45 min vs 7.67 ± 2.02 min, *P* = 0.116) under the fluoroscopy, as well as no significant difference among SPN, mGGO and GGO (6.12 ± 2.05 min, 7.25 ± 1.33 min and 7.80 ± 2.02 min, *P* > 0.05).

**Conclusions:**

Small PPL whether it is visible or not under fluoroscopy can be located accurately by our three-dimensional localization technique on chest wall surface and performed bronchoscopy procedures to increase diagnostic yields. It is more convenient, economical and reliable with the similar diagnostic yields than R-EBUS guided method.

**Trial registration:**

Current Controlled Trials ChiCTR-DDD-16009715. The date of registration: 3rd Nov, 2016. Retrospectively registered.

## Introduction

Nowadays, with the application of high-resolution computed tomography (CT) imaging techniques to lung cancer screening, small solitary pulmonary nodule (SPN) or ground-glass opacity (GGO) lesions in the lung are frequently detected [1, 2]. For this article, the small peripheral pulmonary lesions (PPLs) are defined as lesions less than 3 cm in length, including the SPN, GGO or mixed GGO (mGGO) and being surrounded by the lung parenchyma without evidence of endobronchial abnormalities. The differential diagnosis of PPN is broad, ranging from benign tumors, edema, infectious lesions to lung cancer and other malignant conditions [3]. Lung cancer is the most common cause of cancer death for both men and women with a 5-year global survival rate in patients in the early stages of the disease of 38–67% and in later stages of 1–8% [4]. Thus, the prognosis of lung cancer depends on the early diagnosis of these lesions. When biopsy is recommended, CT-guided transthoracic fine-needle aspiration (TTNA) or biopsy is currently preferred because it has a diagnostic yield of 90%, although perhaps less with smaller lesions [5]. However, TTNA has limitations such as without bronchoalveolar lavage fluid (BALF), brushing, and with respect to the puncture site, seeding along the needle biopsy tract with malignant cells which are potentially serious, with a risk from 0.06 to 1%, as well as a high risk of complications such as a pneumothorax median 25% (range 4–60%), among which 4% (range 0.2–8%) for pneumothorax requiring chest tube and the median risk of hemorrhage is 12% (range 2–66%), and may be higher in the diffuse lung disease patients with bulla under pleura and those with smaller nodules or nodules deep in the lung parenchyma [6, 7]. Another more complex process video-assisted thoracoscopic surgery (VATS), even CT assisted VATS [8] is also an alternative method to obtain the tissue samples especially for some small PPNs. However, sometimes the techniques need an additional tool such as O-arm CT or some preoperative marking procedures to identify the location of the small lesions [9, 10]. For example, VATS involves hook-wire technique, endoscopic ultrasonography, barium marking through bronchoscopy, and percutaneous injections of dyes, colored collagen, lipiodol, agar, and barium, et al. [7]. These methods may also result in some complications mentioned above, which led to its prohibition in Japan [11]. The diagnostic yields of another diagnostic method, flexible bronchoscopy, for PPLs <2 cm in the published reports have varied from 5 to 28% [12]. Therefore, several guided-bronchoscopy technologies have been developed to improve the yields of transbronchial biopsy for PPLs diagnosis, such as real-time MSCT fluoroscopic guidance with higher radiation doses to the patient and operators [13], ultrathin bronchoscope, electromagnetic navigation bronchoscopy (ENB), virtual bronchoscopy (VB), R-EBUS with guide sheath (GS), and lung point [5, 14]. All these methods have improved the diagnostic yields, for example, the overall diagnostic yield of EBUS-GS-guided transbronchial biopsy (TBB) may be 65.0% when the location of the lesion can be confirmed by EBUS [9].

However, in many patients, the location of target lesion is invisible under the x-ray fluoroscopy and yet can not always be identified so precisely by these guided-bronchoscopy technologies and consequentially affect the diagnostic yields. On the other hand, because of the high fee of guided-bronchoscopy equipments and requiring specialized training doctors, the advanced techniques mentioned above are not always available for the hospitals in many developing countries and rural districts.

Therefore, we are studying for a more convenient, economical and reliable method for accurately locating the small PPLs, then perform the bronchoscopy procedures to improve the diagnostic yields. And we also perform R-EBUS with GS for comparison.

## Materials and methods

### About the protocol

The protocol was approved by the University Ethics Committee and the Institutional Review Board of the First Affiliated Hospital of Fujian Medical University and the First Hospital of Longyan City in Fujian Province. Written informed consents prior to performing the procedure were obtained from all the enrolled patients with the indications as follows: PPNs less than 30 mm in diameter size. The patients with the contraindications for the flexible bronchoscopy were excluded. All patients were followed-up until achievements of definitive diagnosis.

### Groups

Patients from the two hospitals from June 2012 to December 2015 were divided into 3 groups. All patients underwent chest CT scanning using a multidetector CT scanner (Aquilion; Toshiba; Tokyo, Japan) prior to bronchoscopy, in order to locate the lung lesions at full inspiration. Images were reconstructed from helical CT data by one radiologist and transferred to a work site. Our new technique was performed on 24 lesions in 24 patients, we also named as fluoroscopy group, who were classified into 2 groups: group1 (*n* = 12) and group2 (*n* = 12) according to the lesion visible or not under fluoroscopy. The lesion visible or not was confirmed by two radiologists. Visible lesion means the lesion can be recognized under fluoroscopy by the both radiologists and invisible lesion means the lesion can not be recognized under fluoroscopy by the both radiologists. However, if the lesion can not be recognized by one, then should be confirmed invisible by the third radiologist. The group 3 was performed EBUS-GS, namely Radial EBUS-GS group.

### Characteristics of patients

Among group 1 and 2, each lung lobe (including left lingular) had lesion for confirming to locate accurately the small PPNs. There were 12 patients with lesion visible under fluoroscopy in the group 1 and also 12 patients with lesion invisible under fluoroscopy in the group 2. There were 23 patients in the group 3. The characteristics of three groups were shown at Table 1.

**Table 1:** Characteristics of three groups

Characteristics	Group 1 (*n* = 12)	Group 2 (*n* = 12)	Group 3 (*n* = 23)
Age (years)	61 ± 18	58 ± 16	60 ± 15
Sex (cases)			
Female	7	6	9
Male	5	6	14
Lesion size (mm)			
Length	25.25 ± 3.05	13.00 ± 6.00	20.50 ± 5.00
Width	14.42 ± 4.81	9.42 ± 3.61	15.00 ± 4.30
Height	16.42 ± 2.31	7.33 ± 2.78	13.00 ± 6.50
Location and cases of each lobe			
Right (left) upper lobe	2	2	6
Right middle (left lingular) lobe	2	2	5
Right (left) lower lobe	2	2	12
CT findings (Types and Cases)			
GGO	0	5	5
mGGO	4	2	6
SPNs	8	5	12

Group 1: Patients with lung lesion visible under fluoroscopy; Group 2: Patients with lung lesion invisible under fluoroscopy; Group 3: Patients performed EBUS-GS

### Bronchoscopy procedures

#### Bronchoscope was advanced to segment where the lesion located

Local anesthesia of the upper respiratory tract was achieved using 2% lidocaine. Continuous pulse oximetry was performed during FB, and blood pressure was measured every 5 min. Oxygen was administered by a nasal cannula, and the flow was adjusted upward from 2 L/min to maintain the pulse oximetric saturation above 90%. After the FB (BF-1T260, Olympus, Tokyo, Japan) was advanced beyond the vocal cords through the nasal route, all segments of the bronchial tree were visualized. Then the FB was advanced to the lesion segment according to the CT scans. FB procedures were performed by well-trained bronchoscopists who had more than 10 years of experience. The average number of FB procedures performed per year by the bronchoscopist was 200 or greater.

#### Visible lesion under fluoroscopy

For the patients with PPN visible under fluoroscopy, fluoroscopy-guided bronchoscopy was performed.

#### Lesions were located accurately by our techniques and performed bronchoscopy procedures

##### Determing core point L of lesion according to crosspoint of three dimensions of lesion

**Fig. 1:**
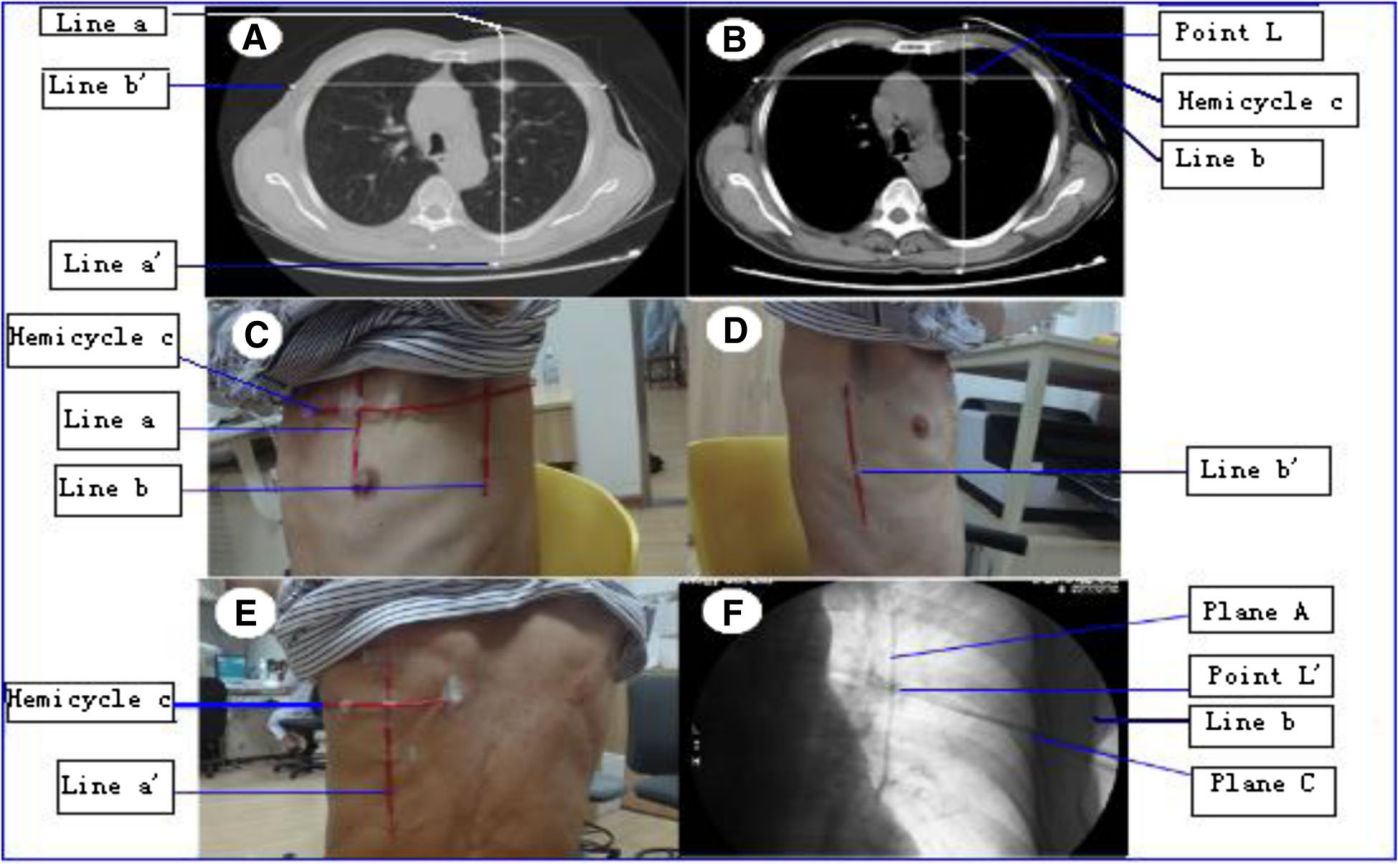
A core point of the lesion which we named as *point L* could be determined according to the crosspoint of the longest diameter of three dimensions, that is length, width and height of the lesion, on CT scans (shown by **a** and **b**). Opaque soft copper wires were respectively placed firmly on the surface of the chest wall with adhesive tape (shown by **c**, **d** and **e**). Under the fluoroscopy, with the careful rotation of the C-arm machine, the crosspoint *L*’ of the three perpendicular planes determined by the three sets copper wires *aa*’, *bb*’ and hemicycle *line c* (shown by **a** and **b** with *bright pots* and *lines*, and by **f** with *dark lines*) on the surface of chest wall could reflect and fit accurately with the *Point L* of the lung lesion (shown by **f**)

All chest CT scans of the enrolled patients were reviewed to detect the longest diameter of three dimensions: length, width and height of the lesion and the characteristics of the all lesions were carefully analyzed. The CT scans mainly aiming at the lesion were reconstructed again with slices of 1 mm under a deep inspiratory breathhold. The bronchi leading to the lesion were also recorded. A core point of the lesion which we named as point L could be determined according to the crosspoint of the longest diameter of three dimensions, that is length, width and height of the lesion, on CT scans (Fig. 1a and b). Especially, if the branches of bronchi leading into the lesion could be recognized, the end point of the bronchi inside the lesion was determined as point L and also was recorded.

##### Point L could be reflected by crosspoint determined by three planes lines on surface of chest wall

**Fig. 2:**
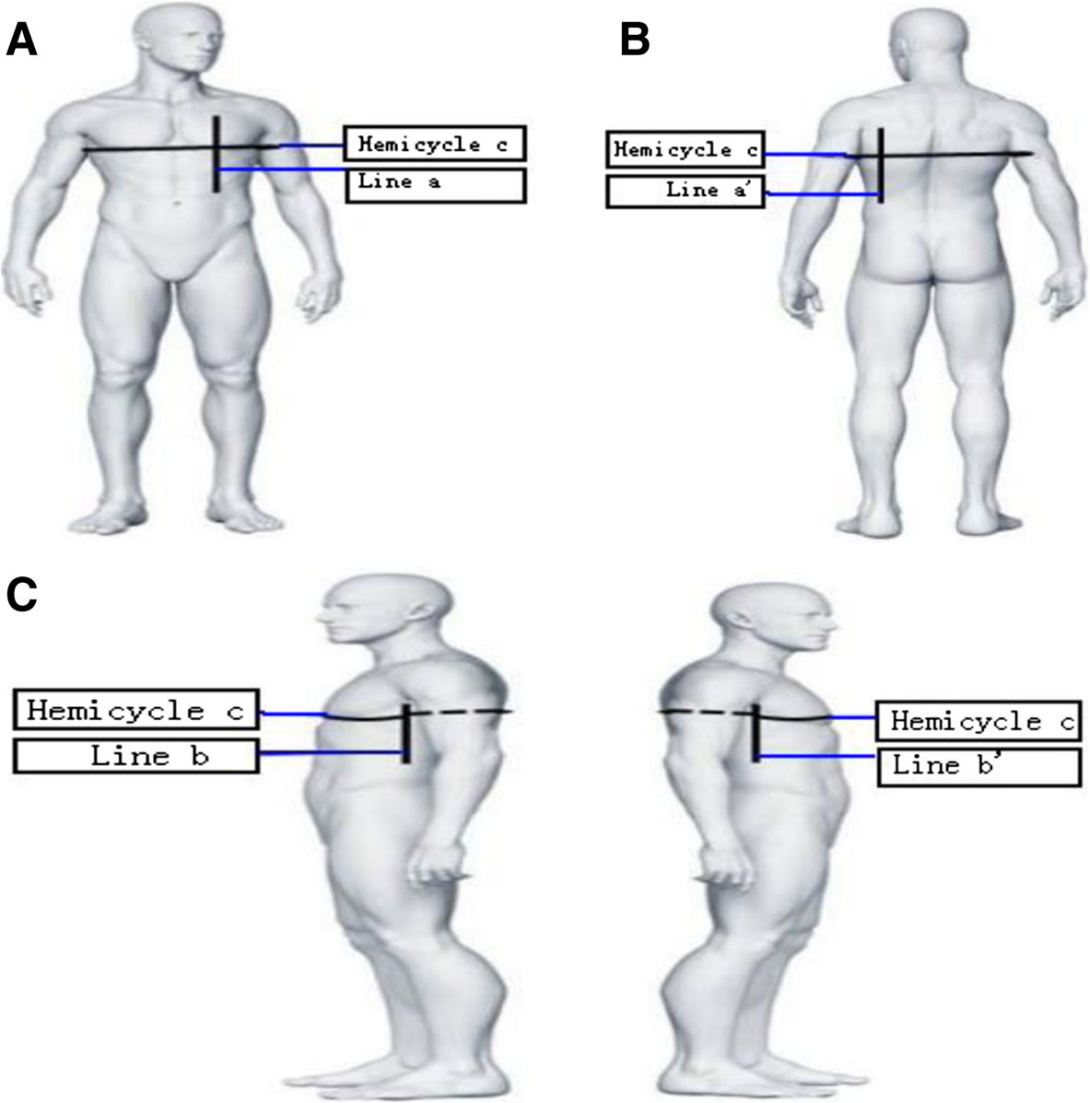
(model): According to the CT parameters scanning the lesion, the three perpendicular lines: *Line a* and *a*’ which determined Sagittal Plane **a**, *Line b* and *b*’ which determined Coronal Plane **b**, and Hemicycle *Line c* which determined Horizontal Plane **c** can be marked on the surface of the chest wall

According to the mathematical principle: Three Perpendicular Planes Intersect at ONE Point, this Point can be reflected onto the surface of the chest wall by the three perpendicular planes lines, that is Sagittal Plane A determined by Line a and Line a’, Coronal Plane B determined by Line b and Line b’ and Horizontal Plane C determined Hemicycle (or Whole Cycle) Line c (Fig. 1a and b). According to the CT parameters scanning the lesion, these three perpendicular planes lines can be marked on the surface of the patients’ chest wall (Clearly shown in Model Fig. 2). Opaque soft wires, such as copper wires which were visible under the fluoroscopy, were respectively placed firmly on the surface of the chest wall with adhesive tape according to the marks (Fig. 1c, d, e). Therefore, although the lung lesion can not be recognized under the fluoroscopy, the another crosspoint we named as L’ of the three perpendicular planes determined by the three sets of copper wires aa’, bb’ and hemicycle line c on the surface of chest wall could reflect and fit accurately with the Point L of the lung lesion (Fig. 1f).

##### Detecting accurately core point L of the lesion under fluoroscopy

Fluoroscopic guidance was provided by a multipurpose C-arm type diagnostic unit (Allura Xper FD20; Philipps Company; Amsterdam, the Netherlands). With the careful rotation of a C-arm unit, we can see the wires a and a’ overlapped together like one wire named as Line A, and intersected with hemicycle c toward the x-ray anteroposterior direction, which meaned that Plane A intersected with Plane C at Line A. Then, going on rotating the C-arm unit, we can also see the wires b and b’ overlapped together like one wire named as Line B, and intersected with hemicycle c toward the x-ray horizontal direction, which meaned that Plane B intersected with Plane C at Line B. As mentioned above, the crosspoint L’ was also the crosspoint of Line A and Line B. Therefore, we should advance the bronchoscope tip into the bronchus toward the crosspoint of Line A and Line B, which reflected the core point L of the lesion under the fluoroscopic guidance.

##### Harvesting specimen focusing around the point L of the lung lesion

The brush or forceps exserting from the bronchoscopy reached the crosspoint L’ under the fluoroscopy. The specimen were harvested focusing around the point L of the lesion through the brushing, forceps biopsy, and BALF.

### ROSE

The rapid on-site evaluation (ROSE) procedure was performed by a cytotechnologist after staining the smears from the tissue obtained during the bronchoscopy, enabling immediate cytopathologic evaluation and feedback regarding specimen adequacy and potential diagnosis. At least 2 smears were prepared, one stained immediately with a rapid stain (Diff-Quik, Baxter Diagnostics, Inc) and the other fixed in alcohol for Papanicolaou (PAP) staining later. Cytologic slides were then sent to the pathologist for immediate interpretation of whether the sample was negative (no malignant cells) or positive (definitive cytopathologic evidence of malignancy) [15]. The remaining specimen was placed in 10% formaldehyde for routine histologic examination and diagnosis.

### Brief case report involving our techniques

**Fig. 3:**
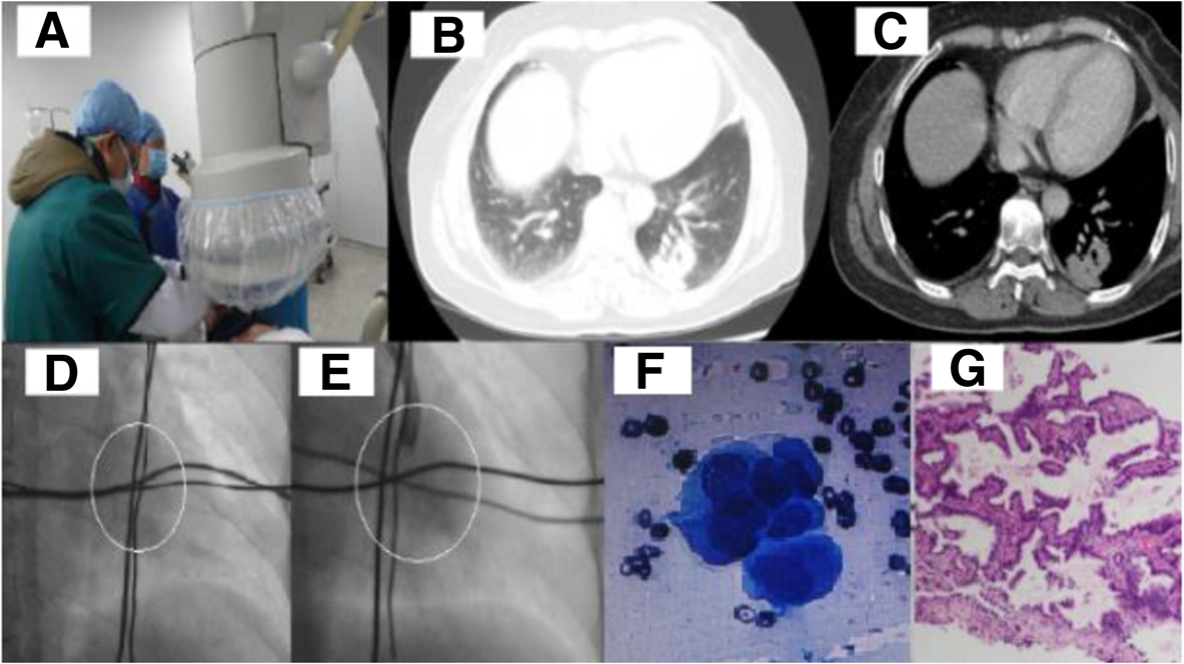
The lung CT showed an irregular shape nodule with 2.0 cm*2.0 cm*1.5 cm size in the left posterior basal segment lobe (**b**, **c**). Although the lesion located behind the heart and was hard to be recognized under the fluoroscopy, the core point of the lesion was detected with the help of our methods of locating lesion (**a**, **d**, shown within the *white circle*). Bronchoscopy procedures under the fluoroscopy were performed to harvest the specimen focusing around the core point of the lung lesion (**d**, **e**, shown within the *white circle*). The pathology from the specimen showed adenocarcinoma (**f**: ROSE, **g**: Histology)

A 75-year-old female with a 5-months’ history of recurrent hemoptysis was admitted to the First Affiliated Hospital of Fujian Medical University. The lung CT showed an irregular shape nodule with 2.0 cm*2.0 cm*1.5 cm size in the left posterior basal segment lobe (Fig. 3b, c). As the principles and techniques mentioned above, the core point of the lesion was determined. Then three sets of metal wires were placed on the surface of the patient’s chest wall. Although the lesion located behind the heart and was hard to be recognized under the fluoroscopy, the core point of the lesion was detected with the help of our methods of locating lesion (Fig. 3a, d). Subsequently, bronchoscopy procedures under the fluoroscopy were done to harvest the specimen focusing around the core point of the lesion (Fig. 3e, f). The pathology from specimen showed adenocarcinoma in situ with immunohistochemisty: TTF-1, CK7, CK19, Napsina(+); PR, CEA(focal+); Tg, Villion, CR, CK5/6, CDX-2, CA125, ER, GCDFP-15, CK20, CA199(−), KI-67(+, 1%) (Fig. 3g).

### EBUS procedures

In the group 3 (patients performed EBUS-GS), equipped with endoscopic ultrasound system (EU-M30S, Olympus, Tokyo, Japan), a 20 MHz radial small-diameter probe (UM-S20–20R Olympus) with guide sheath was inserted through the working channel of the bronchoscope. Once the precise location of the lesion was identified by EBUS, the probe was withdrawn, leaving the GS in place. Subsequently, the specimen were harvested through the brushing, forceps biopsy, and BALF for ROSE and pathologic diagnosis as other groups.

### Statistical analysis

Data were presented as mean ± standard deviation or number (percent) as appropriate. Statistical analysis was performed using *χ*2 test or Fisher’s exact test and *t*-test in SPSS statistical software (SPSS version 15; SPSS; Chicago, IL). A two-sided *p* value of less than 0.05 indicated statistical significance.

## Results

### Results of patients performed bronchoscopy under fluoroscopy

The location of the core point L of the lesion had been determined in the chest CT scans. The point L whether it was visible or invisible under the fluoroscopy could be recognized by the crosspoint L’ of the three perpendicular planes determined by the three sets copper wires aa’, bb’ and hemicycle line c on the surface of chest wall. The results of the patients performed bronchoscopy under fluoroscopy were showed in Table 2.

**Table 2:** Results of patients performed bronchoscopy under fluoroscopy were shown

*N*	Location	CT finding	Size (mm)	Distance to pleura (mm)	Visible or not	Histology results from bronchoscopy	Final diagnosis	Fluoroscopy time (min)
1	RUL	mGGO	30*15*15	15	Visible	Adenocarcinoma	Adenocarcinoma	7.5
2	RLL	SPN	12*10*11	10	Invisible	Negative	Adenocarcinoma	9.0
3	LM(L)L	mGGO	18*15*10	8	Invisible	Inflammation	Inflammation	9.5
4	RLL	SPN	25*15*14	18	Visible	Inflammation?	Adenocarcinoma	7.0
5	RML	GGO	3*4*3	0	Invisible	Negative	Inflammation	10.0
6	LUL	SPN	26*18*16	10	Visible	Caseous necrosis	Caseous necrosis	5.5
7	LUL	SPN	25*11*15	5	Visible	Carcinoma cells	Adenocarcinoma	6.5
8	LLL	SPN	9*8*5	10	Invisible	Negative	AAH	10.0
9	LUL	GGO	4*3*3	15	Invisible	Negative	Inflammation	10.0
10	RML	SPN	25*16*17	22	Visible	Atypical cells	AAH	4.5
11	LULΔ	SPN	10*10*9	15	Invisible	Squamous cell carcinoma	Squamous cell carcinoma	8.0
12	LLL	SPN	20*20*15	0	Visible	Carcinoma cells	Adenocarcinoma	7.5
13	RML	mGGO	22*8*16	10	Visible	Negative	Adenocarcinoma	6.5
14	RUL	SPN	20*8*6	20	Invisible	Squamous cell carcinoma	Squamous cell carcinoma	5.0
15	RUL	mGGO	25*12*16	15	Visible	Negative	Caseous necrosis	5.5
16	RML	GGO	11*13*7	20	Invisible	Carcinomacells	Small cell carcinoma	6.5
17	LM(L)L	mGGO	30*11*20	0	Visible	Poorly differentiated carcinoma	Poorly differentiated carcinoma	7.5
18	RUL	SPN	18*8*10	30	Invisible	Carcinoma cells	Squamous cell carcinoma	4.5
19	RLL	SPN	25*15*22	28	Visible	Small cell carcinoma	Small cell carcinoma	4.0
20	RLL	GGO	19*14*6	30	Invisible	Negative	Squamous cell carcinoma	6.0
21	LLL	SPN	22*10*16	30	Visible	Negative	Inflammation	3.5
22	LM(L)L	SPN	25*12*15	32	Visible	Adenocarcinoma	Adenocarcinoma	4.5
23	LLL*	mGGO	20*10*8	35	Invisible	Carcinoma cells	Adenocarcinoma	7.0
24	LM(L)L	GGO	12*10*10	25	Invisible	Carcinoma cells	Large cell carcinoma	6.5

*N* Number of cases, Visible:lesion can be recognized under fluoroscopy. Invisible:lesion can not be recognized under fluoroscopy. Visible or not: Visible or not under fluoroscopy. Histology results from bronchoscopy include brushing, BALF and biopsies under fluoroscopy. Final diagnosis: histology pathological diagnosis or diagnosis by following up. *RUL* Right Upper Lobe, *RML* Right Middle Lobe, *RLL* Right Lower Lobe, *LUL* Left Upper Lobe, *LM(L)L* Left Lingular Lobe, *LLL* Left Lower Lobe, *SPN* Solitary Pulmonary Nodules, *(m)GGO* (mix) Ground Glass Opacity, *AAH* Atypical Adenomatous Hyperplasia. *:minor hemorrhage at the biopsy site; Δ:pneumothorax not requiring chest tube

### Visible lesion under fluoroscopy

**Fig. 4:**
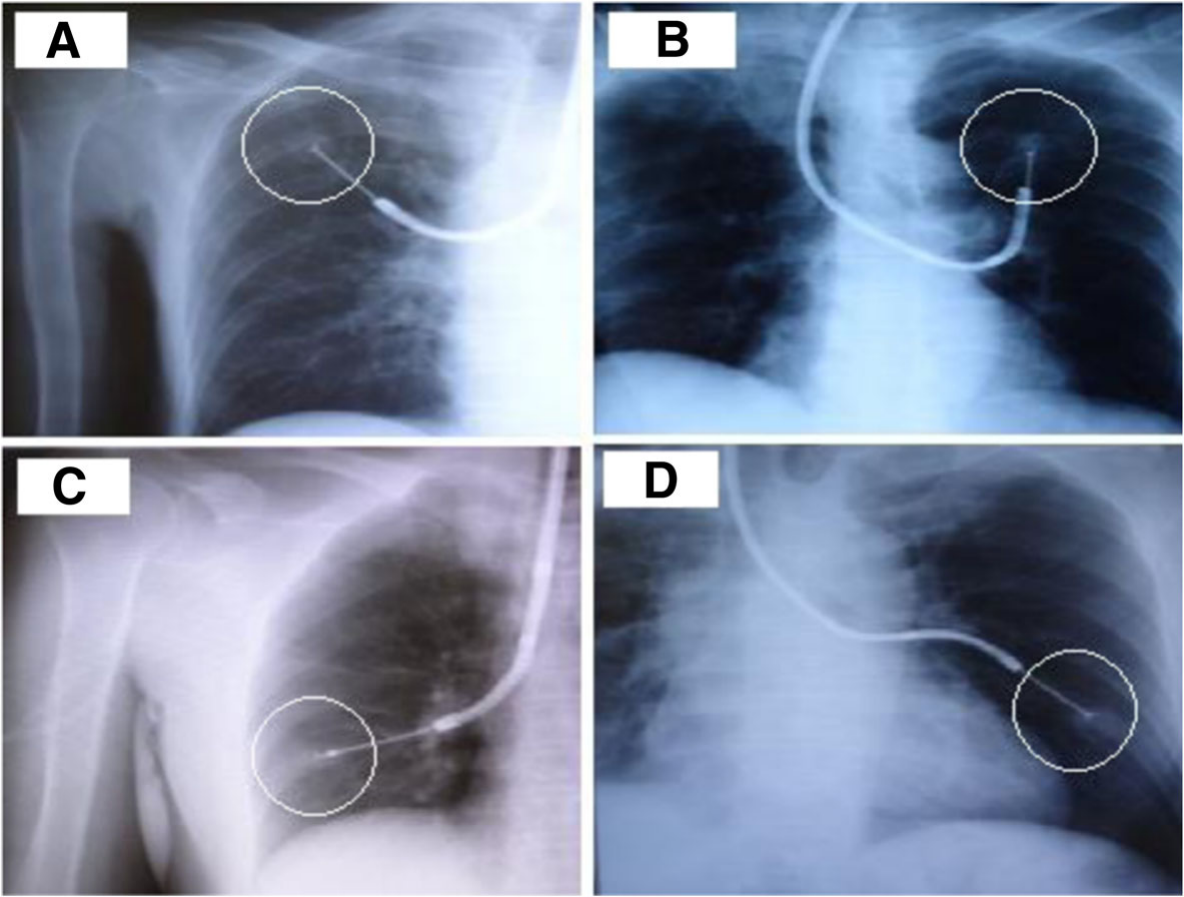
Bronchoscopy procedures focusing on the visible lesion were performed under fluoroscopic guidance according to the lesion location. **a**, Lesion was showed in Right Upper Lobe within circle. **b**, Lesion was showed in Left Upper Lobe within circle. **c**, Lesion was showed in Right Lower Lobe within circle. **d**, Lesion was showed in Left lower Lobe within circle

Fluoroscopy-guided FB was advanced into the bronchus under fluoroscopic guidance according to the lesion location. The brushing, forceps biopsy and BALF focusing on the lesion were performed in these patients (Fig. 4).

### Results of patients performed bronchoscopy with R-EBUS-GS

**Fig. 5:**
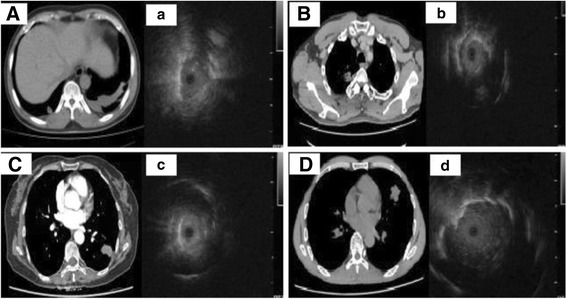
Lesions were detected by the R-EBUS-GS through bronchoscopy according to their locations in different lobes (Right lower lobe shown in **A** and **a**; Right upper lobe shown in **B** and **b**; Left lower lobe shown in **C** and **c**; Left lingular lobe shown in **D** and **d**)

A total of 23 consecutive patients were included in the study, with 9 female and 14 male. The detail characteristics of this group were shown in group 3 in Table 1. The mean age was 60 ± 15 years (range, 47 to 77 years). The average lesion size in length was 20.50 ± 5.00 mm (range, 10 to 30 mm). Lesion was detected by the R-EBUS-GS through FB according to its location (Fig. 5). The accuracy rates of diagnostic yields were 65.2% (15 in 23 cases).

### ROSE results

**Fig. 6:**
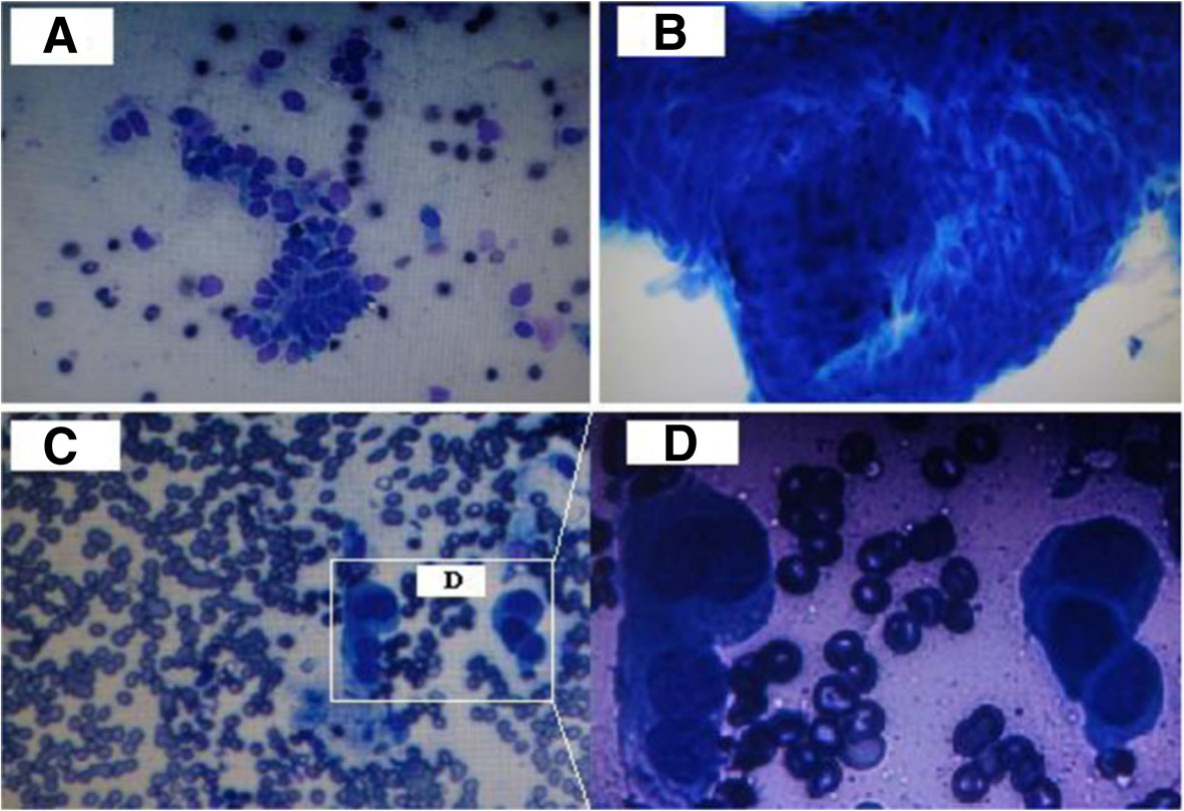
The cancer cells in the ROSE slides may show as: closter (shown by **a**), masslike and overlapped (shown by **b**), scattered (shown by **c** and **d**)

The cancer cells in the ROSE slides may show as:closter, masslike and overlapped, scattered (Fig. 6). Scattered cancer cells in the slides were prone to be ignored. According to whether the biopsy was successful and adequate diagnostic material was harvested from initial specimens, further specimens collection during bronchoscopy (e.g. brushing, biopsy) will be performed or adjusted the biopsy sites [16].

### Accuracy rates of diagnostic cases guided by fluoroscopy guided FB or R-EBUS

The accuracy rates of diagnostic yields were 66.7% in the group 1, 58.3% in the group 2, with average 62.5% in these two groups (both were included in the fluoroscopy group), and was similar as 65.2% in the group 3 (R-EBUS-GS group) (*P* > 0.05). Accurate diagnosis rates were similar among Flu-FB group and EBUS group (15 in 24 patients vs 15 in 23 patients, *P* = 0.846). In the Flu-FB group, there were no significant difference among different CT scan findings (*P* = 0.503) and whether the lesion visible or not under the fluoroscopy (*P* = 0.50). However, in the Flu-FB group, there was a decreasing tendency on accurate diagnosis rates of LL lesions when comparing with non-LL lesions (3/8 = 37.5% vs 12/16 = 75%, *P* = 0.091) while in the EBUS group it was similar (9/12 = 75% vs 6/11 = 54.6%, *P* = 0.278). And in the EBUS group, there was a significant difference among different CT scan findings (*P* = 0.044) with SPN vs GGO (*P* = 0.028) and whether the lesion detectable or not by EBUS (*P* = 0.008) (Table 3).

**Table 3:** Accuracy rates of diagnostic cases guided by fluoroscopy guided FB or R-EBUS

Methods Cases Items		Lesion location			CT scan findings			Lesion under fluoroscopy or EBUS	
		UL	ML	LL	SPN	mGGO	GGO	Visible	Invisible
Flu-FB	Cases enrolled	8	8	8	13	6	5	12	12
	Accurate diagnosis	6	6	3	9	4	2	8	7
EBUS	Cases enrolled	6	5	12	12	6	5	19	4
	Accurate diagnosis	3	3	9	10	4	1	15	0

*UL* Upper lobe, *ML* Middle lobe, *LL* Lower lobe. Lesion under fluoroscopy or EBUS

### Average fluoroscopy time during bronchoscope procedures

In the fluoroscopy group (Flu-FB group), the average fluoroscopy time was (6.75 ± 1.96) min. There were no significant difference of the fluoroscopy time in lower lobe patients compared with the not lower lobes patients ((6.75 ± 2.24) min vs (6.75 ± 1.88) min, *P* > 0.05). The length of the visible lesion is much larger than the invisible lesion (25.00 ± 2.92 mm vs 13.00 ± 6.00 mm, *F* = 8.844, *P* = 0.007). Fluoroscopy time was negatively correlated with the lesion length (*r* = −0.613, *P* = 0.001). However, about fluoroscopy time, there was no significant difference between visible lesion and the invisible lesion (5.83 ± 1.45 min vs 7.67 ± 2.02 min, *P* = 0.116), as well as no significant difference among SPN, mGGO and GGO (6.12 ± 2.05 min, 7.25 ± 1.33 min and 7.80 ± 2.02 min, *P* > 0.05).

### Complications

There was 1 patient with the complication of minor hemorrhage and 1 patient with pheumothorax not requiring chest tube in group 2. No complication was found in other groups.

## Discussion

### All of lung lesions shown by chest CT scans can be successfully localized by our techniques

Lung cancer is currently the leading cause of cancer deaths worldwide. The mortality rates have remained high because of the difficulty in detecting early stage of this disease. Fortunately, with the wide application of HRCT, PPLs are frequently detected which made early diagnosis become possible. However, using CT characteristics alone to determine the lesions as benignity, premalignancy or malignancy is often difficult [17]. The tissue samples of the PPNs can be harvested by the FB. The location of the lesion should be determined before harvesting the samples, thus can improve the diagnostic yield. The lung lesion location is estimated by chest radiograph (CXR) or CT of the chest. It is a challenge to determine the location of the small lesion, especially which is invisible under the fluoroscopy.

However, whether the lung lesion is visible or not under the fluoroscopy. The core point of the lesion detected by CT scans can be reflected by the crosspoint of three planes determined by the three sets of opaque soft wires placed on the surface of chest wall. According to the mathematical principle: Three Perpendicular Planes Intersect at ONE Point, the lesion can be located accurately by our techniques. Then, under the fluoroscopy, the bronchoscopy procedures can be performed aiming at the crosspoint of the three perpendicular planes and the specimen can be harvested by focusing around the point, which is also the core point of the lung lesion. This technique does not make use of sophisticated equipments, and it is easy to be understood and mastered.

### Performing bronchoscopy by our three-dimensional localization technique

#### Accuracy rates of diagnosis by bronchoscopy whether lesion is visible or not under fluoroscopy are similar

For non-endobronchial peripheral lung lesion, if the lesion is visible under fluoroscopy, the brush and biopsy forceps of the bronchoscopy can be advanced into the bronchus under fluoroscopic guidance according to the lesion location, as been successfully performed in our study. According to Rittirak et al. study, the overall diagnostic yield of Flu-TBLB group may be statistically significantly higher than NFlu-TBLB group (43.8 vs. 32.9%; *P* = 0.003) [18]. However, the diagnostic yields of FB for peripheral pulmonary lesions <2 cm in published reports have varied from 5 to 28%, because some lesions are invisible through conventional flexible bronchoscopy under fluoroscopy [12].

In our study, although lung lesion is invisible under fluoroscopy, we can recognize it by the crosspoint of three perpendicular planes determined by the opaque soft copper wires placed on chest wall surface, therefore, the diagnostic yields have been improved, which is similar as R-EBUS-GS group. And this location method will not be influenced by the different size, whether the lesion visible or not under the fluoroscopy and CT scan characteristics of the lesion, such as SPN, mGGO or GGO.

#### Accuracy rates of diagnosis by bronchoscopy of non-lower lobe lesion are higher than lower lobe lesion

However, due to the respiratory motion, mainly caused by the diaphragm motion, the lung lesion may be mislocalized [19]. Movement of the lung occurs with respiratory variation during bronchoscopy, and the location of pulmonary nodule during procedures may differ significantly from its location on the initial planning, especially to the lower lobe, which moved significantly more than upper lobe lesion. In the Flu-FB group of our study, a decreasing tendency has been observed on accurate diagnosis rates of LL lesions when comparing with non-LL lesions, while in the EBUS group it was similar. Actually, one of the main challenges for us is hard to find the exact position to perform a biopsy for the lesion located in the lower lobe when respiratory motion. According to the other studies, this movement during bronchoscopy may even significantly affect the diagnostic yield of electromagnetic navigation bronchoscopy procedures [20]. The lesion size and distance from the pleura doesn’t significantly impact the movement. Holding a deep breath may manage the respiratory motion problem, but the duration of bronchoscopy procedures which involve BALF, biopsy, brushing et al., may take some longer time. The navigation system with the proposed respiratory motion compensation method and EBUS allow for real time guidance during bronchoscopic interventions, and thus could increase the diagnostic yield [21]. Therefore, combing with other diagnostic techniques may improve diagnostic yields.

### Diagnostic yield about R-EBUS

The technique of R-EBUS with a GS has definitely its place in the diagnostic work-up of PPLs, especially for the lesions that can be visualized by EBUS. The diagnostic yield has fluctuated from 53% up to 77% [22], which can be increased by ensuring that TBB is performed at the correct location as identified by the EBUS. Tay et al. performed a retrospective analysis of 196 consecutive patients who underwent investigation with R-EBUS [23]. They found that a definitive diagnosis was established for 109 PPL (56%) using radial EBUS. Visualized lesion by EBUS probe had a higher diagnostic yield (65%) than EBUS-invisible lesions (20%; *P* = 0.0001) and in multivariate analysis, lesion size, malignancy status and distance from hilum to lesion were significant predictors of EBUS visualization yield. In our study, lesions performed R-EBUS were visualized by EBUS probe and there were same diagnostic yields as Tay et al’s study. On the other hand, different CT scan findings have been associated with different diagnostic yields bronchoscopy guided by R-EBUS-GS, especially high with SPN lesion and low with GGO lesion. However, the overall diagnostic yields by R-EBUS-GS were similar with that by our three-dimensional localization technique according to our study. What we are concerned about is that the diagnostic yield of EBUS-invisible lesions may be improved according to our three-dimensional localization technique.

### CT-guided transthoracic fine-needle aspiration

TTNA or biopsy is widely used currently for its high diagnostic yields [5]. However, TTNA has a limitation mentioned in the introduction part. Tai et al. has evaluated the frequency and severity of pulmonary hemorrhage after transthoracic needle lung biopsy (TTLB) and assess possible factors associated with pulmonary hemorrhage [24]. Tai et al. has found that the pulmonary hemorrhage occurred in 483 of the 1175 TTLBs (41.1%) but rarely requires intervention and higher-grade hemorrhage was more likely to occur with female sex (*P* = 0.001), older age (*P* = 0.003), emphysema (*P* = 0.004), coaxial technique (*P* = 0.025), nonsubpleural location (*P* < 0.001), lesion size of 3 cm or smaller (*P* < 0.001), and subsolid lesions (*P* = 0.028). However, to the lower lobe lesion, because of low accuracy rates of diagnosis by our techniques, we recommend to perform CT-guided TTNA or combine with other motheds to improve the diagnostic yield.

### Role of ROSE

As we know, ROSE of tissue obtained by FNA has been first introduced by Washington University investigators in St. Louis, Missouri. Its major advantages have been improvements in health care resource utilization, safety, reduction in the number of sites biopsied, allowing immediate processing and interpretation of the sample in the procedural suite, lowering patient costs, increase of sample adequacy rates and diagnostic yield, decrease x-ray exposure time during radiologically guided interventional procedures and other potential morbidity related to the biopsy procedure [25–28], which has been reported to have 85 to 93% sensitivity and 100% specificity [29]. ROSE can also aim at molecular profiling (optimal lung cancer genotyping) in patients with advanced lung cancer [30]. Therefore, it is recommended to be performed routinely, especially in patients with a suspected diagnosis of lung cancer [31]. In our study, ROSE has been performed in the 3 groups and the cancer cells were found to be mainly distributed as :1) closter, 2) masslike and overlapped, and 3) scattered. Scattered cancer cells in the slides were prone to be ignored. Therefore, the scattered characteristics of cancer cells should be paid much more attention to. However, the main reason for the scarcity of performing ROSE in other practices is oftenly the lack of on-staff cytopathologists [32].

### Fluoroscopy time in our study

It takes appropriated procedure time to perform bronchoscope under fluoroscopy. There are a negative correlation between the fluoroscopy time and the lesion length. However, because the lesions can be located accurately by our techniques and performed bronchoscopy procedures, there is no significant difference on fluoroscopy time between visible lesion and the invisible lesion, as well as no significant difference among SPN, mGGO and GGO. Fluoroscopic guidance may be also utilized in many other bronchoscopic procedures, including EBUS for investigation of PPLs. Therefore, x-ray exposure during radiologically guided interventional procedures should be taken into consideration including the medical staffs and patients. The fluoroscopy time in our study was defined as the time from the lesion was nearly reached by the bronchoscope to the biopsy time point. With the help of ROSE in our study, the fluoroscopy time is some shorter, comparing with Fujita Y et al’s study [4]. However, Müller et al. reported that the duration of fluoroscopy time correlated with the radiation dose to the hands of the surgeons, and that radiation dose without thyroid protection was 70 times higher than with thyroid protection in a comparison [33]. Therefore, the reduction of fluoroscopy time and proper shielding of radiation doses are recommended.

### Adverse effects

It is very convenient for us to use fluoroscopy to check for adverse effects of pneumothorax and hemorrhage, to confirm the position of the brush and forceps with opening or not. Factors that were associated with the increased risk of pneumothorax include patient characteristics (age >60 years; COPD), lesion characteristics (small size, greater distance from pleura), and technical factors (increased number of punctures or aspirates, biopsy performed in supine or lateral rather than prone position). In our study, as one patient with pneumothorax and one patient with minor bleeding, complications do exist [34].

### It is recommended to combe with many diagnostic techniques for increasing diagnostic yields

Many advanced bronchoscopy techniques have been studied and performed in the patients with PPL, but as clinicians, we should take these factors into considerations: the choice of optimal investigation, determinants of diagnostic yield for bronchoscopy, complications, and practice pattern variations [34]. As mentioned above, the accuracy of CT-guided lung biopsy is high. However, lesions that are smaller or deeper in the lung result in a higher number of CT scans with increased radiation dose and procedure time, seeding along the needle biopsy tract with malignant cells and the lesions surrounding emphysema, lung bullae or pulmonary vascular, whic are at high risk for pneumothorax or hemorrhage following TTNA. Introducing the biopsy through the bronchus within the target lesion increases the diagnostic yield and also decreases the risk of pneumothorax [35, 36 ]. When it is more difficult for the biopsy needle to hit the lesion during CT-guided biopsy, it is more likely for specimens to be retrieved and diagnosed from the BALF and brushing guided by the brochoscopy with the help of our three-dimensional localization technique on chest wall surface. On the other hand, measuring and applying the distance between the orifice of bronchus and the lesion could increase the diagnostic yield of EBUS-guided TBBs for PPLs [36]. According to our results, to non-lower lobe PPLs, performing bronchoscopy by our three-dimensional localization technique may be an optional selection, especially its convenient and economical. And to lower lobe, we prefer to perform EBUS-GS procedures.

### Limitations of our study

Firstly, the number of the patients is not enough. Secondly, x-ray exposure to the medical staffs and patients during radiologically guided interventional procedures should be taken into much consideration, and the fluoroscopy time may be shorter after our proficiency on performing the related procedures, for example performing the ROSE and manipulating the C-arm unit. Thirdly, there is no data about the combination ulities of diagnostic methods, for example, three-dimensional localization technique plus EUBS or/and CT-guided TTNA or biopsy. Further studies focusing on these limitations are needed.

## Conclusions

Small PPNs invisible under fluoroscopy can be located accurately by our three-dimensional localization technique on chest wall surface and performed bronchoscopy procedures to increase diagnostic yields. It is a more convenient, economical and reliable method with the similar diagnostic yields as R-EBUS guided method. This localization technique can also be performed in other organs such as the deep lesion of pancreas etc. The ulities of multiple diagnostic methods are recommended to increase diagnostic yields of PPLs according to the equipments and specialized training available in the hospitals.

## Abbreviations

BALF: Bronchoalveolar lavage fluid
CXR: Chest X ray
ENB: Electromagnetic navigation bronchoscopy
FB: Flexible bronchoscopy
Flu: Fluoroscopy
GGO: Ground-glass opacity
GS: Guide sheath
LL: Lower lobe
mGGO: Mixed GGO
PPLs: Small peripheral pulmonary lesions
R-EBUS: Radial endobronchial ultrasound
ROSE: Rapid on-site evaluation
SPN: Small solitary pulmonary nodule
TBB: Transbronchial biopsy
TTLB: Transthoracic needle lung biopsy
TTNA: Transthoracic fine-needle aspiration
VATS: Video-assisted thoracoscopic surgery
VB: Virtual bronchoscopy
